# Microscopy of the Dental Tissues

**Published:** 1869-05

**Authors:** S. P. Cutler


*To be Continued.*

---

### ARTICLE. II.

### *Microscopy of the Dental Tissues. New Series.*

By S. P. Cutler, M.D.,A.E.G.,D.D.S,

*Continued.*

Histology and Physiology—It will be seen by formulas given in the previous article, that fats and oils are the most important calorifacients found in the organism except free phosphorous and sulphur. Let us examine into Creative wisdom in the distribution of these elements. Fat is found in healthy animals just under the skin over the whole body, in thick sheets as the omentum covering the abdominal vis ciru, studding the mesentery, covering portions of the heart and kidneys, and filling the hollows of long bones except in birds whose bones are filled with warm air and very light. It is always found where most needed as heat generator and non-conductor, also to fill places unoccupied by other tissues, and serves mechanical purposes as well as vital. The cavities of the chest and cranium are not protected to the same extent as the abdomen by fat, as it is not necessary they should, notwithstanding these cavities contain the three vital organs. These cavities are enclosed by bony non-conducting walls, one being the hemal and the other neural, the hemal being the principal aerating surface, the mural being the centre of sensation, inteligence and volition. The latter in consequence is more highly endowed with the more energetic oxygen consumer phosphorus, superceding the necessity of the more cumbrous agent fat, and here space is important. We may then suppose that the greatest amount of heat generating material is deposited where most needed, and this same supposition may equally apply to all other elements existing in the organism. Climatic influence in relation to dermal and sebaceous endowments is very great. Animals in cold climates require thicker and heavier fur and hair, and a greater amount of fat, as well as more food of combustion to resist the radiating influence of cold from the lungs and skin. Birds are more exposed to such influences, have a higher temperature and require more fat and dermal raiment as feathers, and more food, and starve to death sooner than mammals. Cold blooded animals as Reptiles and cold blooded Fishes have but little surplus fat. The warm blooded Cetaceans breath air and have large deposits of fat on the surface and require more food. A snake can live six months on one frog, as their nutrition and waste is exceedingly slow, as their bodies do not resist external changes of temperature to any great extent. Animals that attain a definite size in a given time have a definite limit of existence, and if not removed by disease or accident perish by natural limitation. As these limits can never be extended or transcended they may be regarded as definite and determinate, they may, by indiscretion, abuse and violation of hygienic and physiological laws, shorten the limitation allotted to each individual existance, when by proper observance of these laws the ultimate limits may be reached, which may vary in each individuality. In childhood and youth the elements are oxidizable, owing to excess of oxidizable elements and a corresponding activity of oxidation, nutrition and growth, all the tissues are more oily and less dense than in more advanced years.

Cell contents are more oxidizabl than cell structures, there being scarcely any vital resistance offered in the former case to the action of oxygen. Oxidation here is not meant putrefaction which does not take place in the living body in living tissues, such however being the case after death in a part or whole—hence oxidation taking place in a living body is different from that which takes place after vital resistance ceases. In fasting in fevers and old age, there is less of nutrition in fevers, there is increased oxidation in fasting, and in old age there is diminished nutrition and oxidation both,and less capability of resisting the influence of external changes —not so in fevers. In all the above cited cases the first to suffer oxidation is cell contents throughout the entire organism, though in different degrees in different structures.

In all the above cases there is diminished energy in the symptoms, owing to loss of normal vitality, though brought about somewhat differently. As old age advances, the cells and contents become less oxidizible. the tissues becoming more dead, less vital resistance, less energy, the chemical forces proper gradually gain on the vital until they are completely subordinated and death ensues. The body even then is capable of being trans-substantiated into other living organisms. As old age advances the same bulk and weight may be retained as in middle life, still, histology teaches us that there have been losses in certain elements and immediate principles, the change being in favor of the inorganic disconnected from the organic kingdom by a loss of balance in the forces of oxidation and nutrition, or decay and reproduction. In the burning of starch, sugar, and oils the presence of azotised compounds may play a catalytic part by converting oxygen into ozone, consequently fermentation of the tirnary groups; there is however no fermentive action, formation of alcohol and carbonic and other acids, and farther on acetic acid instead, only carbonic acid and water being formed, no splitting up into one inorganic and another equivalent of organic division as in ordinary fermentations of the tirnary group—outside of the animal organism the process is different. The vital properties of muscles consist exclusively in contractility by shortening and widening, not by any indwelling power inherent in the muscular fibres themselves, not an instrinsic but an extrinsic power or force sent to them from points outside, not by any demand being made by the muscles themselves, they being wholly passive and unconcious until suddenly roused by this outside stimuli or vis-a-tergo being forced in by motor nerves. The muscles in themselves are similar to the bones, only passive agents obedient to some other outside influence. Respiration, circulation and voluntary motion are but muscular motion that motion being an effect secondary to something else called vital force and dwells in the brain and nervous system, this in turn is secondary being but a reserve force received from all points in the organism wherever oxidation takes place. The power and duration of muscular contraction depends on due development of muscular and neural systems and hygienic conditions. Each cell forms its own equivalent of vital force and sends it up to general headquarters on one set of nerves, and is returnd by another set of nerves, the sensorial and motors. The brain is a charged prime conductor, while the passive muscle is the negative conductor, so to speak, and when the charge is turned loose by volition it is by the same act directed to a certain muscle or muscles, causing them to contract, they in turn then become positive to the brain which becomes negative, until another act of volition turns off the charge from the muscle causing it to relax, then the order is again reversed. There are certain tissues that possess very low vital properties simply chemica-vegetative, and only perform passive functions in the organism, as bones, ligaments, tendons, fascias, and others still are no less important in making up the "*tout ensemble*" of life. The blood though a moving tissue possesses vital properties of the highest order, is propelled by intrinsic andextrinsic force, both the intrinsic may be supposed to be polar force, dependent on polarity of the corpuscles among themselve and polar relations, between the moving mass and the tissues, the extrinsic force being muscular or propelling force. All life forces probably are derived from or are resident in that fluid, and perform the most important primary functions to the entire organism, the brain and nervous systems performing secondary functions. The blood is the primum mobile of the organism. As already stated the nerves are but factors, on the one hand for the cells and on the other for the brain, or carriers of forces, sometimes termed vital, sometimes magnetic, nervous electric, or vis vitæ, *and so on.*

The brain is the leyden jar holding statical force, bottled up as it were, ready to be turned to use by act of the will upon the voluntary muscles causing contractions.

The nerves of involuntary motion are not under the direction of the will to any great extent, but are excited to action by the moving current of the blood, stimulating the heart and arteries by friction and oxidation causing magnetic and thermal disturbances sufficient to excite the nerves presiding over this system. The blood rushing to the lungs may stimulate the nerves of respiration, also independent of the will, both being sustained by duto dynamical battery, while volition is sustained by a statical battery, both contained in the brain and nervous system, each independent of the other so far as their special functions are concerned. When these forces are once spent on muscular contraction, as relaxation follows this force is dissipated or rendered inoperative, and may be of no further use in the economy, so far as we know, or it may be converted into some other force and again utilized in some way—who can say? As these forces pass along the motor canals there is no perceptible disturbance whatever in them, no sign or manifestation until muscle is reached where its power is spent, which causes muscular contraction and relaxation.

During muscular contraction there is an increase of circulation in the muscle, and a corresponding increase of oxidation and nutrition and a greater amount of force generated, though itself imparts no additional power to the muscular act until it is sent up to the great nerve centres and returned again through the motor nerves to the part. As muscles contract they swell laterally, but not in proportion to the amount of shortening, in consequence there must be a certain amount of condensation, this may be proved by their rigidity during contraction. Our enquiry now leads to what is muscular contraction? We can imagine muscular polarity in the walls of muscular fibres themselves, by turning from a lineal to a lateral of 90 degrees so as to bring the poles of two together, thereby shortening in proportion to the relative length and thickness of each molecule or groups of molecules.

In such cases the will makes and breaks the connection or polarity in voluntary muscles. Another theory might be given with some plausibility, that is that the will or nerve force, when sent to the muscle, drives out for the time being some other force or power of cohesion substituting a more subtle and finer force thereby occupying less space in consequence permitting the nearer approach of molecules by this substitution of forces; and whenever the will force is withdrawn relaxation takes place. We may also suppose the nerve force to be the more energetic of the two, but occupying less space among the molecules, but an increase of cohesive force or power, something similar to that of expanding and contracting of metals by changes of temperature alone, which determines the relative amount of cohesive force in such bodies which is directly as the mass and inversely as the square of the distances of atoms. Whatever muscular contraction may be, it is magnetic molecular disturbance no doubt, and what has been said of voluntary equally applies to involuntary motion of muscles, the difference being in batteries used, one statical the other duto dynamic, the force when in motion being the same, producing the same results on muscles, one acting rythmatically with marked regularity the other irregularly and spasmodically governed by caprice of will. What has been said of normal action may equally apply to abnormal or spasmodic action, one physiological the other pathological, owing to unnatural disturbance in the forces and unusual application of them overcoming the will.

There is one other hypothesis I will endeavor to account for, muscular contractility. As the sun passes the equator it acts as a disturbing cause on the otherwise static electricity, sending currents at an angle of 90 degrees towards the poles; it is this force that causes the needle to point north and south. Now, we will imagine the will or brain to act as a disturbing cause and send magnetic forces along the motor nerves longitudinally through the muscular fibres, *the same as the sun,*

directing currents laterally on all sides at an angle of 90 degrees causing cells in opposite fibrils to attract each other from about their centre bringing them in contact, and at the same time mechanically shortening and widening the cells; also a condensation of molecules gives rigidity to the mass; each cell would be shortened and widened, the transverse divisions of the cells being brought nearer together. The nuclei may be regarded as points of positive and negative polarity, and points of attraction.

The magnetic circles in the organism are established by the opposite sets of nerves as connected with the brain and meeting at their distal points.

The proto-dynamics originate in the cells caused by oxidation, also in oxidation of the moving fluids. All forces are economised and adjusted in the nicest and most accurate manner, so as not to suffer any unnecessary loss in the complex organisms of animal life.

*To be Continued.*

---

